# Global, regional, and national burden of elderly myocarditis (1992–2021) and projections of future disease burden trends

**DOI:** 10.1007/s40520-025-02979-9

**Published:** 2025-03-24

**Authors:** Weichun Wang, Xiaofeng Chen

**Affiliations:** Department of Cardiology, The Second Affiliated Hospital of Fujian Medical University, Quanzhou, 362000 China

**Keywords:** Geriatric myocarditis, Age standardized incidence rate, Disease burden, DALYs, Deaths

## Abstract

**Objective:**

Geriatric myocarditis represents a significant public health concern, directly influencing overall health and potentially leading to various cardiac diseases. This study seeks to quantify the burden of geriatric myocarditis over three decades (1992–2021) and provide forecasts for future disease burden.

**Methods:**

Data on geriatric myocarditis from 1992 to 2021 was obtained from the Global Burden of Disease study, offering insights into the incidence of the condition, categorized by gender. The Joinpoint regression model was utilized to identify shifts in epidemiological trends, while decomposition analysis helped identify the underlying factors contributing to these trends. To project future incidence, deaths and DALYs (Disability-Adjusted Life Years), the Norpred and Bayesian Age-Period-Cohort (BAPC) models were employed.

**Results:**

In 2021, the global ASIR (per 100,000) of elderly myocarditis was 47.57 (27.52–73.08), with 505,147 (292,319–774,561) cases. Age-standardized deaths(per 100,000) were 2.07 (1.55–2.51), totaling 20,718 (15,525–25,085) deaths, and age-standardized DALYs(per 100,000) were 29.77 (22.60–35.81), with 308,101 (234,226–370,674) DALYs. Greenland, Canada, and Austria had the highest ASIR(per 100,000), while Romania, Kazakhstan, and Croatia had the highest age-standardized deaths(per 100,000), and Romania, Kazakhstan, and Guyana had the highest age-standardized DALYs(per 100,000). Joinpoint Regression analysis revealed a recent upward trend in global incidence after a previous decline, consistent across genders and SDI regions. Deaths and DALYs showed declining trends globally, though male deaths recently increased. Decomposition analysis identified population growth and aging as key drivers of increased cases, deaths, and DALYs. Based on the nordpred model, by 2045, the global ASIR(per 100,000) is predicted to be 47.27, with 1,005,593 cases, age-standardized deaths(per 100,000) of 2.02, totaling 48,501 deaths, and age-standardized DALYs(per 100,000) of 26.21, with 595,694 DALYs. The BAPC model predicts a global ASIR(per 100,000) of 51.82, with 1,091,195 cases, age-standardized deaths(per 100,000) of 3.67, totaling 87,145 deaths, and age-standardized DALYs(per 100,000) of 49.09, with 1,084,738 DALYs.

**Conclusion:**

As of 2021, the ASIR(per 100,000) of myocarditis in the elderly population showed a decline compared to 1992; however, a recent upward trend has been identified. Considering ongoing population growth, the number of myocarditis cases among the elderly is anticipated to increase.

**Supplementary Information:**

The online version contains supplementary material available at 10.1007/s40520-025-02979-9.

## Introduction

Myocarditis is a condition characterized by injury to the myocardium resulting from a variety of factors [1]. Common causes include viral infections, abnormal immune responses, drug reactions, and autoimmune diseases [2]. The risk of myocarditis in the elderly is heightened by several factors, including a decline in immune system function, the presence of comorbidities such as hypertension and diabetes, and the potential long-term use of certain medications[3]. These patients may exhibit symptoms including heart failure, arrhythmias, chest pain, and dyspnea. If these symptoms are severe, they can result in heart failure or other complications, significantly increasing the risk of mortality[4]. However, in some instances, the symptoms of myocarditis in elderly patients may be more subtle or atypical than those observed in younger individuals, which can lead to a higher likelihood of them being overlooked [5]. Due to the ambiguous nature of symptoms in geriatric myocarditis and the presence of comorbidities such as hypertension and diabetes, early diagnosis and prompt treatment are essential [6].

While myocarditis affects individuals across all age groups, its impact on the geriatric population is particularly concerning due to the physiological vulnerabilities associated with again [7]. Older adults often present with atypical symptoms, complicating prompt diagnosis and treatment. Despite its clinical significance, geriatric myocarditis remains understudied, and its public health burden is frequently underestimated. Recent studies have highlighted the increasing prevalence of myocarditis among older adults, driven by factors such as heightened susceptibility to infections, age-related immune dysregulation, and comorbidities like diabetes and hypertension[8]. Moreover, the aging global population underscores the urgency of addressing this issue, as the burden of myocarditis in older adults is expected to rise in tandem with demographic shifts [9].

However, the lack of comprehensive data on the epidemiological trends of geriatric myocarditis hampers efforts to develop targeted interventions. For instance, while global trends indicate a decline in deaths and DALYs attributed to myocarditis, emerging evidence suggests a concerning rise in mortality among male patients in recent years [10]. These disparities highlight the need for greater awareness, improved diagnostic strategies, and tailored treatment approaches for this vulnerable population. Simultaneously, analyzing the epidemiological trends of myocarditis in elderly patients and implementing targeted preventive measures in high-incidence areas are critical for reducing the incidence of myocarditis within this population [11]. This study aims to quantify the burden of geriatric myocarditis over a 30-year period (1992–2021) and provide predictions for future disease burden.

## Methods

### Materials

The 2021 Global Burden of Disease Study (GBD 2021) offers a comprehensive estimation of geriatric myocarditis incidence rates [12]. These estimates are derived from the GBD 2021 study, available through the GBD Results Viewer at Https://vizhub.healthdata.org/gbd-results/, with the latest access recorded on December 26, 2024. The data sources for geriatric myocarditis encompass a diverse array of records from multiple countries, including hospitalization data, emergency room visits, insurance claims, surveys, and vital registration systems [13].

The research utilized anonymized data compiled by the Institute for Health Metrics and Evaluation (IHME) at the University of Washington [14]. The study protocol, including the waiver of informed consent, was reviewed and approved by the Institutional Review Board (IRB) of the University of Washington. The 2021 Global Burden of Disease Study (GBD 2021) offers a comprehensive analysis of 369 diseases and injuries, along with 87 associated risk factors, thoroughly examined across 204 countries and territories. The GBD research team has outlined their methodological approach and published ASIR(per 100,000) estimates for these health conditions. The methodology employed in GBD 2021 adheres to the principles outlined in the 11th edition of the ICD, wherein each death is attributed to the underlying cause that initiated the sequence of events leading to death. In cases where vital registration data reported ICD codes classified as "garbage codes" (a term used in GBD to denote non-specific codes, implausible codes, or codes indicating intermediate rather than underlying causes of death), these were reassigned to the most probable cause of death using redistribution algorithms. These algorithms were developed based on published studies, expert consultations, or regression analyses applied to data sources reporting multiple causes of death [15].

To enable comparative analysis, the Global Burden of Disease project categorizes countries and regions according to their Socio-Demographic Index (SDI). This index classifies nations into five distinct tiers: high SDI (> 0.81), upper-middle SDI (0.70–0.81), middle SDI (0.61–0.69), lower-middle SDI (0.46–0.60), and low SDI (< 0.46). This classification framework emphasizes both the similarities and disparities in epidemiological patterns across various countries and regions [16].

### Socio-demographic index

The Socio-Demographic Index (SDI) is a comprehensive metric that aggregates social and economic variables to assess their impact on health outcomes across different regions [17].It is derived by calculating the geometric mean of three constituent elements: the total fertility rate for individuals under 25 (TFU25), the mean educational level attained by those aged 15 and older (EDU15 +), and the per capita income distributed over time (LDI). In the GBD 2021 study, SDI values were scaled up by a factor of 100 to generate a new range from 0 to 100 (further details can be found at Https://ghdx.healthdata.org/record/global-burden-disease-study-2021-gbd-2021-socio-demographic-index-sdi-1950–2021).

### Statistical analysis

Firstly, the incidence rates of geriatric myocarditis for eight age groups (60–64, 65–69, 70–74, 75–79, 80–84, 85–89, 90–94, 95 +) by gender were extracted from the GBD database. The ASIR(per 100,000) of geriatric myocarditis were subsequently calculated based on the incidence rates for these age groups. A pyramid chart was utilized to visualize the incidence rates by gender across the various age groups. Additionally, a world map was employed to illustrate the ASIR(per 100,000) by gender globally for the years 1992 and 2021.

Joinpoint regression analysis was performed using Joinpoint software to identify inflection points in the trends of geriatric myocarditis incidence. Decomposition analysis was conducted to uncover the driving factors behind the changes in the epidemiological trends of geriatric myocarditis. The Slope Index of Inequality (SII) and Concentration Index (CI) were applied to assess the existence of gender-related health inequities in global geriatric myocarditis incidence in 2021. The Nordpred model and Bayesian age-period-cohort (BAPC) models were employed to predict the ASIR(per 100,000) and the number of geriatric myocarditis cases for the next thirty years, extending to 2046, by gender.

Demographic projections used in this study were derived from the Global Burden of Diseases (GBD) 2017 database. All analytical procedures were conducted using R software (version 4.3.3, developed by the R Core Team in Vienna, Austria), with statistical significance established at a P-value threshold of less than 0.05.

## Results

### Global burden analysis from 1992 to 2021

**Fig. 1:**
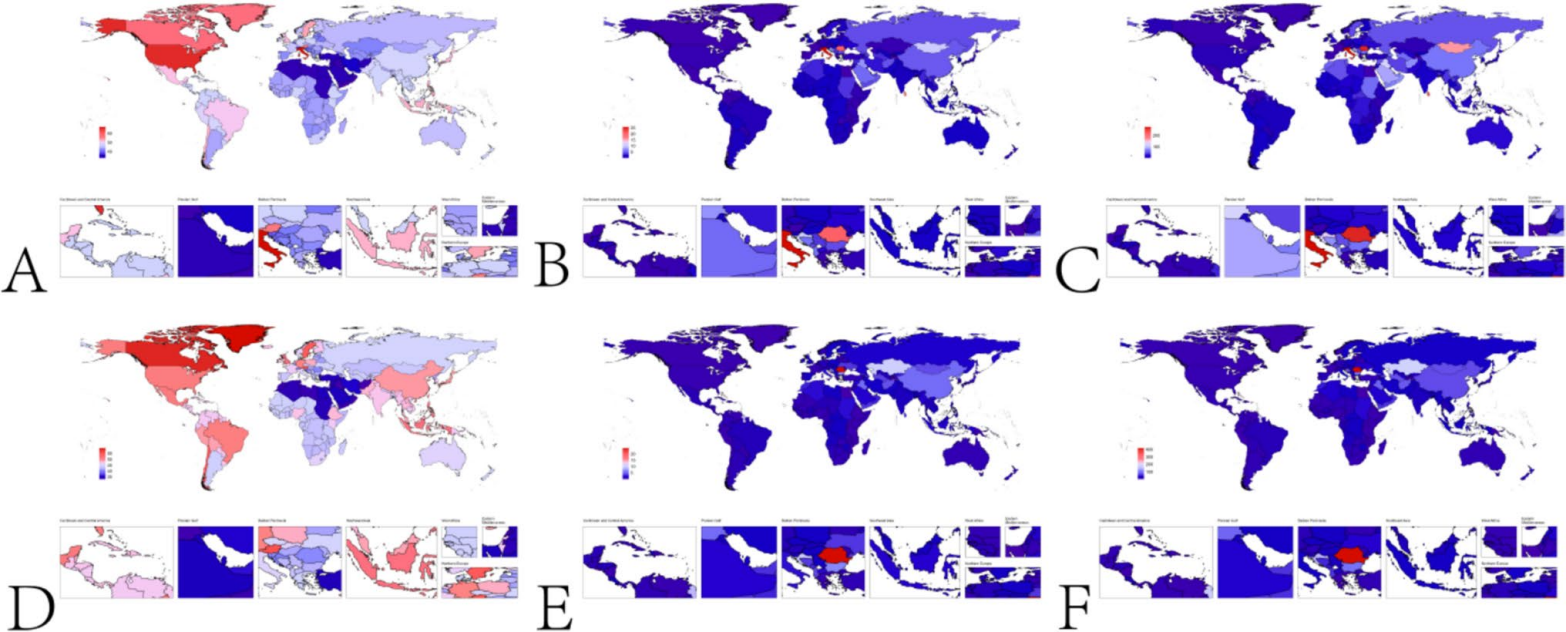
Global incidence of myocarditis in the elderly in 1992 and 2021. **A**. ASIR(per 100,000) of global elderly myocarditis in 1992. **B**. Age-standardized deaths(per 100,000) from global elderly myocarditis in 1992. **C**. Age-standardized DALYs(per 100,000) for global elderly myocarditis in 1992. **D**. ASIR(per 100,000) of global elderly myocarditis in 2021. **E**. Age-standardized deaths(per 100,000) from global elderly myocarditis in 2021. **F**. Age-standardized DALYs(per 100,000) for global elderly myocarditis in 2021

**Fig. 2:**
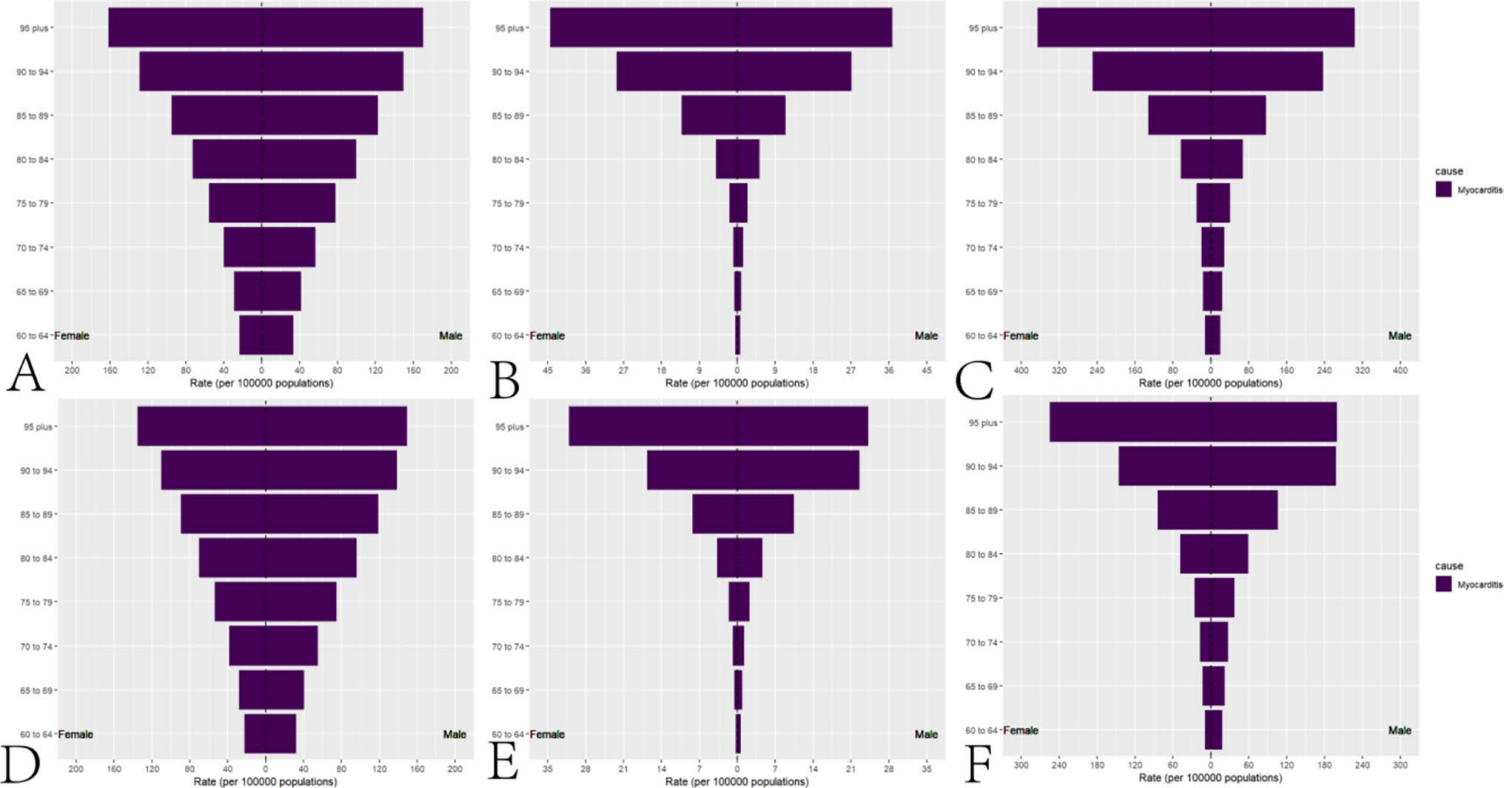
Global incidence of myocarditis in the elderly by gender in 1992 and 2021. **A**. ASIR(per 100,000) of global elderly myocarditis by gender in 1992. Age-standardized deaths(per 100,000) from global elderly myocarditis by gender in 1992. **C**. Age-standardized DALYs (per 100,000) for global elderly myocarditis by gender in 1992. **D**. ASIR(per 100,000) of global elderly myocarditis by gender in 2021. **E**. Age-standardized deaths(per 100,000) from global elderly myocarditis by gender in 2021. **F**. Age-standardized DALYs(per 100,000) for global elderly myocarditis by gender in 2021

**Fig. 3:**
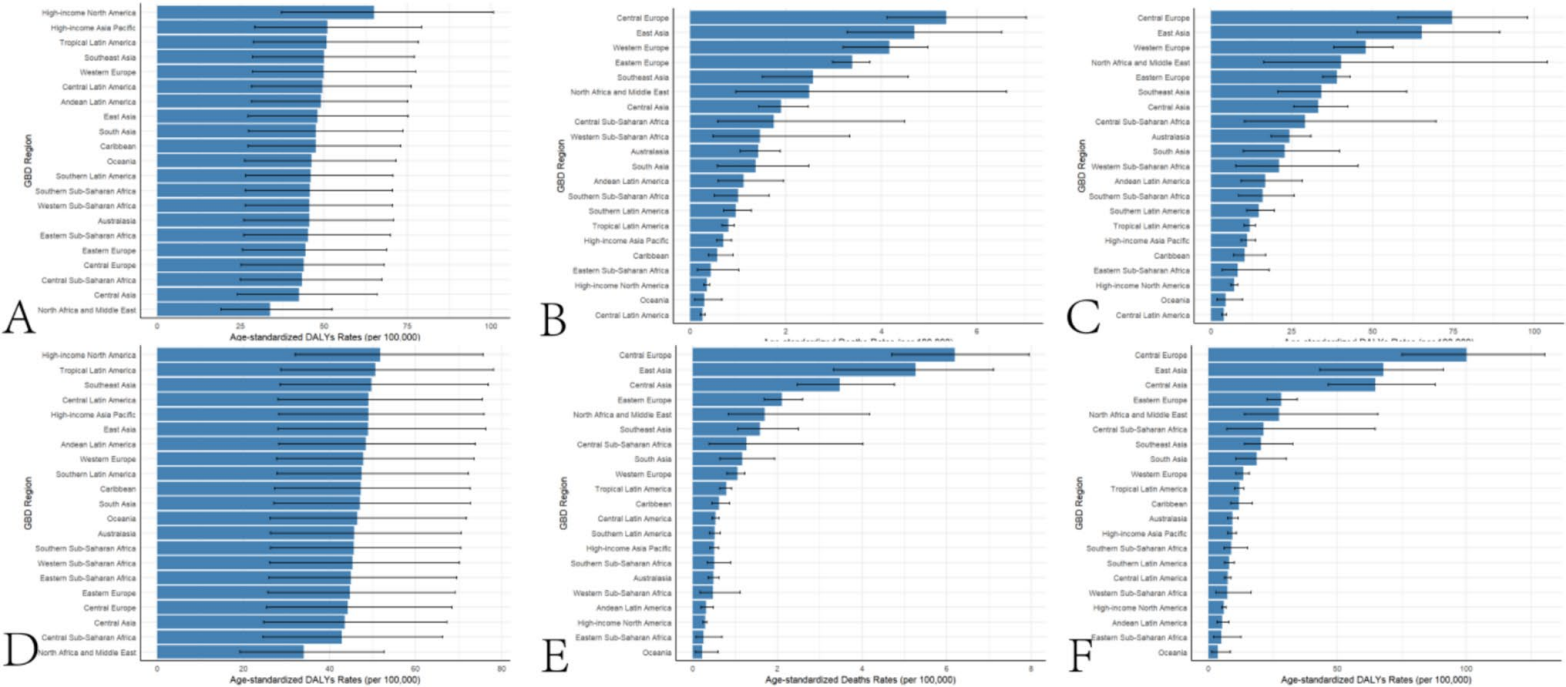
Global incidence of myocarditis in the elderly by SDI region in 1992 and 2021. **A**. ASIR(per 100,000) of global elderly myocarditis by SDI region in 1992. **B**. Age-standardized deaths(per 100,000) from global elderly myocarditis by SDI region in 1992. **C**. Age-standardized DALYs(per 100,000) for global elderly myocarditis by SDI region in 1992. **D**. ASIR(per 100,000) of global elderly myocarditis by SDI region in 2021. **E**. Age-standardized deaths(per 100,000) from global elderly myocarditis by SDI region in 2021. **F**. Age-standardized DALYs(per 100,000) for global elderly myocarditis by SDI region in 2021

In 1992, the global ASIR(per 100,000) of myocarditis in the elderly was 49.29 (28.26–76.43), with a total of 232,688 (133,805–358,397) cases. Among males, the ASIR(per 100,000) was 58.43 (33.56–90.56), with 119,272 (68,793–183,022) cases, while among females, the ASIR(per 100,000) was 42.44 (24.22–65.63), with 113,415 (64,854–175,395) cases. The global age-standardized deaths(per 100,000) from myocarditis in the elderly in 1992 were 2.70 (2.14–3.27), with a total of 10,162 (8,062–12,399) deaths. Among males, the age-standardized deaths(per 100,000) were 2.77 (2.13–3.63), with 4,218 (3,181–5,745) deaths, while among females, the age-standardized deaths(per 100,000) were 2.60 (1.95–3.25), with 5,943 (3,181–5,745) deaths. The global age-standardized disability-adjusted life years (DALYs, per 100,000) for myocarditis in the elderly in 1992 were 37.01 (29.27–45.37), with a total of 157,497 (124,549–195,100) DALYs. Among males, the age-standardized DALYs(per 100,000) were 39.96 (30.29–53.97), with 73,380 (54,723–102,391) DALYs, while among females, the age-standardized DALYs(per 100,000) were 34.15 (25.40–43.23), with 84,116 (62,382–107,172) DALYs. In 2021, the global ASIR(per 100,000) of myocarditis in the elderly was 47.57 (27.52–73.08), with a total of 505,147 (292,319–774,561) cases. Among males, the ASIR(per 100,000) was 56.29 (32.58–86.57), with 267,270 (154,925–408,877) cases, while among females, the ASIR(per 100,000) was 40.46 (32.58–86.57), with 237,877 (366,054–137,146) cases. The global age-standardized deaths from myocarditis in the elderly in 2021 were 2.07 (1.55–2.51), with a total of 20,718 (15,525–25,085) deaths. Among males, the age-standardized deaths(per 100,000) were 2.41 (1.71–3.11), with 9,905 (7,005–12,823) deaths, while among females, the age-standardized deaths(per 100,000) were 1.81 (1.27–2.32), with 10,812 (7,553–13,802) deaths. The global age-standardized DALYs(per 100,000) for myocarditis in the elderly in 2021 were 29.77 (22.60–35.81), with a total of 308,101 (234,226–370,674) DALYs. Among males, the age-standardized DALYs(per 100,000) were 35.57 (25.43–45.88), with 160,116 (114,441–207,295) DALYs, while among females, the age-standardized DALYs(per 100,000) were 24.99 (17.84–31.87), with 147,984 (105,596–188,696) DALYs. Supplementary Table S1 presents the incidence, deaths, and DALYs of myocarditis in the elderly globally and across different Socio-demographic Index (SDI) regions. Supplementary Table S2 provides the incidence of myocarditis in the elderly by country and region. Supplementary Table S3 details the deaths from myocarditis in the elderly by country and region, and Supplementary Table S4 outlines the DALYs for myocarditis in the elderly by country and region. Figure 1 illustrates the global incidence, deaths, and disability-adjusted life years (DALYs) of myocarditis in the elderly for the years 1992 and 2021. Figure 2 presents the incidence, deaths, and DALYs of myocarditis in the elderly across different age groups globally in 2021. Figure 3 displays the incidence, deaths, and DALYs of myocarditis in the elderly across different Socio-demographic Index (SDI) regions for the years 1992 and 2021. In 2021, among all countries and regions, Greenland (59.73, 34.32–92.04), Canada (58.89, 34.78–90.86), and Austria (55.53, 33.15–83.50) had the highest ASIR(per 100,000) of elderly cardiomyopathy, while Palestine (33.07, 17.74–51.28), Afghanistan (33.15, 18.78–51.48), and Lebanon (33.15, 18.78–51.48) had the lowest ASIR(per 100,000) of elderly cardiomyopathy. In 2021, among all countries and regions, Romania (24.61, 34.78–34.12), Kazakhstan (9.93, 5.84–15.35), and Croatia (9.89, 6.81–13.59) had the highest age-standardized deaths(per 100,000) from elderly cardiomyopathy, while Cook Islands (0.00, 0.00–0.00), Tajikistan (0.02, 0.01–0.04), and Egypt (0.03, 0.00–0.14) had the lowest age-standardized deaths(per 100,000) from elderly cardiomyopathy. In 2021, among all countries and regions, Romania (418.02, 289.88–584.77), Kazakhstan (180.24, 109.20–272.73), and Guyana (161.54, 106.04–234.01) had the highest age-standardized DALYs (per 100,000) for elderly cardiomyopathy, while Niue (0.01, 0.00–0.01), Tokelau (0.01, 0.00–0.03), and Cook Islands (0.02, 0.01–0.04) had the lowest age-standardized DALYs(per 100,000) for elderly cardiomyopathy.

## Analysis of disease trends

**Fig. 4:**
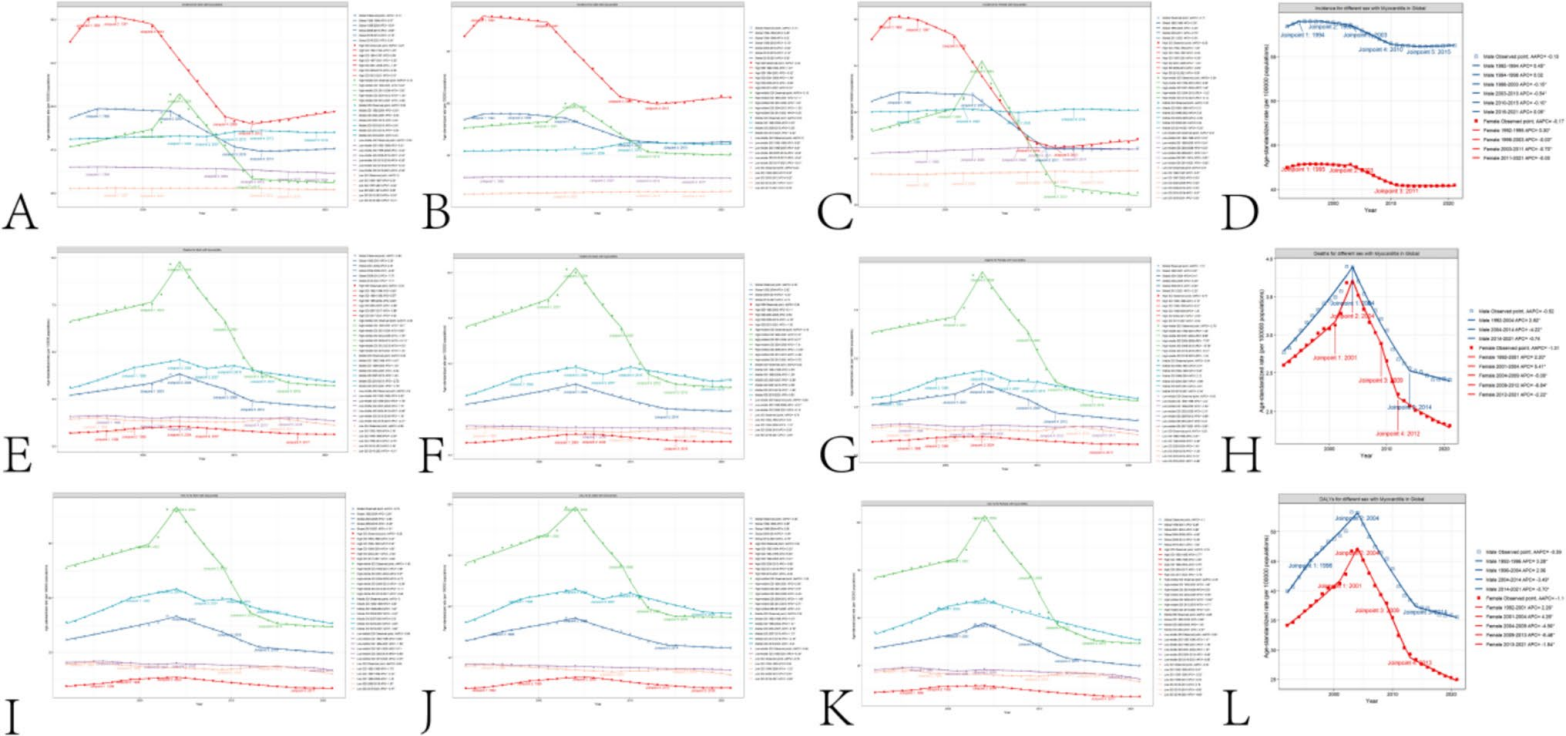
A. Global epidemiological trends in the Incidence of senile myocarditis. **B**. Global epidemiological trends in the Incidence of senile myocarditis among elderly males. **C**. Global epidemiological trends in the Incidence of senile myocarditis among elderly females. **D**. Global epidemiological trends in the Incidence of senile myocarditis among both elderly males and females. **E**. Global epidemiological trends in Deaths due to senile myocarditis. **F**. Global epidemiological trends in Deaths due to senile myocarditis among elderly males. **G**. Global epidemiological trends in Deaths due to senile myocarditis among elderly females. **H**. Global epidemiological trends in Deaths due to senile myocarditis among both elderly males and females. **I**. Global epidemiological trends in DALYs due to senile myocarditis. **J**. Global epidemiological trends in DALYs due to senile myocarditis among elderly males. **K**. Global epidemiological trends in DALYs due to senile myocarditis among elderly females. **L**. Global epidemiological trends in DALYs due to senile myocarditis among both elderly males and females

Through Joinpoint Regression analysis, it has been observed that the global incidence of elderly myocarditis previously exhibited a declining trend, but in recent years, there has been an upward trend. This phenomenon is evident in both male and female elderly populations and is consistent across all SDI regions worldwide. It is worth noting that the incidence of elderly myocarditis in high SDI areas has shown an increasing trend in recent times, regardless of gender. The incidence of elderly myocarditis in the other four SDI regions has remained relatively stable in recent years. Globally, there has been a declining trend in DEATHS due to senile myocarditis, but there has been a recent upward trend in DEATHS among male patients due to the condition. The global trend for DALYs caused by senile myocarditis has also been decreasing, both for males and females. The epidemiological trends of Incidence, Deaths, and DALYs for senile myocarditis among both males and females globally are largely parallel. The results of the Joinpoint Regression are illustrated in Fig. 4 and Table S5.

## Decomposition analysis

**Fig. 5:**
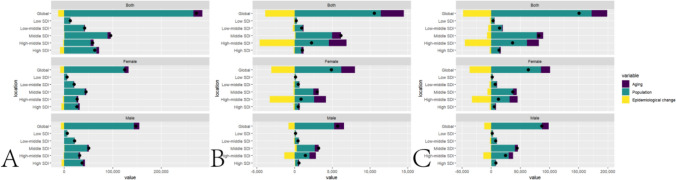
A. Decomposition analysis of global Incidence of senile myocarditis. **B**. Decomposition analysis of global Deaths due to senile myocarditis. **C**. Decomposition analysis of global DALYs due to senile myocarditis

Based on the results of the decomposition analysis, population growth and aging are identified as the driving factors behind the increase, deaths and DALYs in the number of cases of elderly myocarditis globally, with population growth being the primary driver. The results of the decomposition analysis are presented in Table S6 and Fig. 5.

## Prediction of future incidence rate

**Fig. 6:**
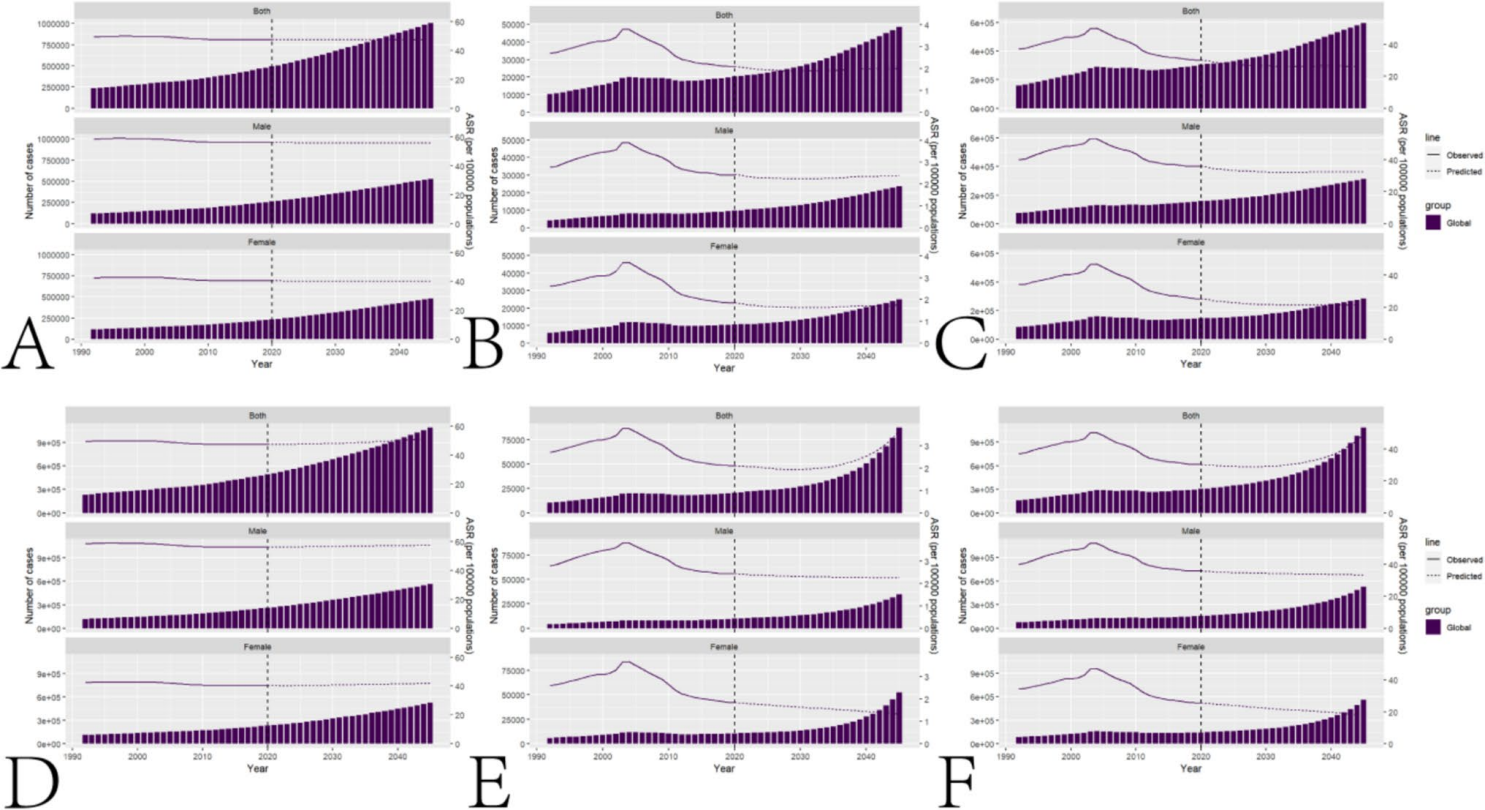
A. Prediction of future global Incidence of senile myocarditis based on the nordpred model. **B**. Prediction of future global Deaths due to senile myocarditis based on the nordpred model. **C**. Prediction of future global DALYs due to senile myocarditis based on the nordpred model. **D**. Prediction of future global Incidence of senile myocarditis based on the BAPC model. **E**. Prediction of future global Deaths due to senile myocarditis based on the BAPC model. **F**. Prediction of future global DALYs due to senile myocarditis based on the BAPC model

Based on the nordpred model, it is predicted that by 2045, the global ASIR(per 100,000) of senile myocarditis will be 47.27, with a total number of cases reaching 1,005,593. Among these, the ASIR(per 100,000) for males is projected to be 55.85, with a total of 528,007 cases, while the ASIR(per 100,000) for females is expected to be 40.06, with a total of 477,586 cases. According to the nordpred model, the global age-standardized deaths(per 100,000) due to senile myocarditis are predicted to be 2.02, with a total of 48,501 deaths. Specifically, the age-standardized deaths(per 100,000) for males are forecasted to be 2.39, totaling 23,621 deaths, and for females, 1.73, totaling 24,880 deaths. The model also predicts that the global age-standardized DALYs(per 100,000) for senile myocarditis will be 26.21, with a total of 595,694 DALYs. For males, the age-standardized DALYs(per 100,000) are expected to be 32.28, with a total of 311,820 DALYs, and for females, 21.20, with a total of 283,874 DALYs. Based on the BAPC model, predictions for 2045 indicate that the global ASIR(per 100,000) for senile myocarditis will be 51.82, with a total number of cases reaching 1,091,195. The ASIR(per 100,000) for males is projected to be 57.57, with a total of 566,024 cases, while the ASIR(per 100,000) for females is expected to be 41.80, with a total of 525,171 cases. The BAPC model forecasts that the global age-standardized deaths(per 100,000) due to senile myocarditis will be 3.67, with a total of 87,145 deaths. Specifically, the age-standardized deaths(per 100,000) for males are predicted to be 2.24, totaling 34,549 deaths, and for females, 1.34, totaling 52,596 deaths. Additionally, the model predicts that the global age-standardized DALYs(per 100,000) for senile myocarditis will be 49.09, with a total of 1,084,738 DALYs. For males, the age-standardized DALYs(per 100,000) are expected to be 33.20, with a total of 527,075 DALYs, and for females, 17.95, with a total of 557,663 DALYs.These projections for the future incidence of elderly myocarditis are visually summarized in Fig. 6.

## Discussion

In our study, we noted that the ASIR(per 100,000) of myocarditis among the elderly population in 2021 showed a slight decline compared to 1992, although the change was not statistically significant. In contrast, the absolute number of myocarditis cases among the elderly increased markedly in 2021. Significant improvements in global hygiene conditions and medical standards have occurred since 1992. With these advancements, it has been established that various factors—such as viral infections, bacterial infections, drug toxicity reactions, radioactive cardiac damage, and myocardial ischemia—contribute to the risk of myocarditis in the elderly[18]. After a virus invades the human body, it triggers an immune response and an inflammatory reaction as a result of the replication process, which can lead to damage to myocardial cells[19].Bacteria can either directly invade myocardial tissue or provoke immune responses that lead to myocardial injury[20]. As health awareness increases, some elderly individuals have started to consciously engage in exercise to boost their immunity and enhance their resistance to pathogens such as bacteria and viruses, thereby lowering their risk of developing myocarditis[21]. Following a viral or bacterial infection, it is advised that elderly patients get adequate rest and avoid overexertion to prevent the onset of myocarditis[1]. Additionally, some dietary supplements are believed to have potential in preventing myocarditis. However, there are currently no effective methods established for the prevention of myocarditis[22]. As a result, the ASIR (per 100,000) of myocarditis in the elderly population has not shown significant changes.

In our study, we found that since 1992, the ASIR(per 100,000) of myocarditis in the elderly population has been steadily declining; however, a recent upward trend has emerged. This change may be associated with the COVID-19 pandemic. COVID-19 itself carries a risk of myocarditis[6], and there have also been reports suggesting that vaccination may trigger myocarditis[23]. To facilitate early diagnosis, it is advisable to consider cardiac magnetic resonance imaging for patients[24]. Relative fat mass (RFM) as an abdominal obesity criterion for metabolic syndrome can be used to identify metabolic syndrome as a risk factor for senile myocarditis[25]. The observed increase in myocarditis incidence among the elderly in recent years may be partially influenced by the COVID-19 pandemic, which has introduced new risk factors for myocardial injury[26]. COVID-19-associated myocarditis, caused directly by SARS-CoV-2 infection, has been well-documented and is thought to result from viral invasion of myocardial cells, systemic inflammation, and immune-mediated damage[27]. Studies have shown that SARS-CoV-2 can induce myocarditis through mechanisms such as cytokine storm, endothelial dysfunction, and direct viral cytotoxicity, particularly in elderly patients with pre-existing cardiovascular conditions[28].

To identify the primary driving factors behind the sharp increase in the incidence of myocarditis among the elderly population, this study employed a decomposition analysis approach. The results revealed that aging and population growth are the key contributors to the substantial rise in myocarditis cases among older adults. The pyramid chart illustrated that the incidence of myocarditis escalates with age. Our study highlights the significant role of population aging and growth in the increasing burden of myocarditis among the elderly. However, it is essential to consider the impact of age-related comorbidities and risk factors that were not included in the decomposition analysis but are critical to understanding the pathophysiology of myocarditis in this population. Conditions such as heart failure, atrial fibrillation, and chronic kidney disease (CKD) are highly prevalent in the elderly and may exacerbate the risk of myocarditis through mechanisms such as chronic inflammation, oxidative stress, and hemodynamic instability[29]. Additionally, autoimmune diseases, which are more common in older adults, can contribute to myocarditis by promoting immune-mediated myocardial injury[30]. Medications commonly used in the elderly population, such as immune checkpoint inhibitors, certain antibiotics, and vaccines, have also been associated with myocarditis[31]. For example, immune checkpoint inhibitors, widely used in cancer therapy, can trigger immune-related adverse events, including myocarditis, due to their mechanism of action. While these factors were not included in the decomposition analysis, their potential contribution to the observed trends in myocarditis cannot be overlooked. Future studies should aim to incorporate these comorbidities and medication exposures into predictive models to better understand their impact on myocarditis risk and outcomes in the elderly population. As the population ages, the increasing number of older individuals further contributes to the rising incidence of myocarditis within this demographic. However, population growth has emerged as the most significant driving factor for the pronounced increase in myocarditis cases among the elderly, more so than aging itself. Looking ahead, continued global population growth is anticipated, which may further elevate the incidence of myocarditis among the elderly[32]. We employed two predictive models to forecast the incidence rate of myocarditis over the next thirty years. The projections indicate that by 2046, the ASIR(per 100,000) of myocarditis among the elderly is expected to range from 47.26 to 57.54. Specifically, elderly males are predicted to have an incidence rate between 52.09 and 55.83, while elderly females are projected to have a rate between 40.04 and 41.77. Moreover, it is anticipated that by 2046, the total number of myocarditis cases among the elderly will range from 1,028,778 to 1,121,817. In this demographic, elderly males are expected to account for 539,963 to 581,263 cases, whereas elderly females are projected to account for 488,815 to 540,554 cases. Given these projections, it will be essential to enhance the monitoring of cardiac function in the elderly and to ensure early diagnosis of myocarditis in this population to improve health outcomes[33].

This study utilizes data from the Global Burden of Disease (GBD) study, which provides high-quality estimates. However, it also has certain unavoidable limitations. First, while the GBD study categorizes data by country and region, it lacks demographic information on race, potentially overlooking the influence of racial factors on the incidence of myocarditis in the elderly. Second, data from earlier years or from countries and regions with lower levels of development may be less accurate due to limited representation and reliance on estimated data derived from various methodologies. Additionally, the GBD registry relies on forecast analysis, which may introduce uncertainties, particularly in regions with incomplete or sparse data. Furthermore, while the Nordpred and BAPC models used in this study are well-established for trend analysis and forecasting, they have inherent limitations, such as their reliance on historical data and assumptions about future trends. These models may not fully capture complex, non-linear relationships that could be better addressed by advanced machine learning or AI-driven predictive analytics. Future studies could explore the integration of AI-based methods, such as deep learning or ensemble models, to enhance the accuracy and robustness of myocarditis risk stratification and forecasting. These limitations should be taken into account when interpreting the results of this study.

## Conclusion

In 2021, the ASIR (per 100,000) of myocarditis in the elderly population showed a decline compared to 1992, but a recent upward trend has been observed. With population growth, the number of myocarditis cases among the elderly is expected to continue rising.

## Supplementary Information

Below is the link to the electronic supplementary material.

Supplementary file1 (XLSX 73 KB)

## Data Availability

No datasets were generated or analysed during the current study.
